# Viable chimaeric viruses confirm the biological importance of sequence specific maize streak virus movement protein and coat protein interactions

**DOI:** 10.1186/1743-422X-5-61

**Published:** 2008-05-20

**Authors:** Eric van der Walt, Kenneth E Palmer, Darren P Martin, Edward P Rybicki

**Affiliations:** 1 Department of Molecular and Cell Biology, University of Cape Town, Cape Town, South Africa; 2 James Graham Brown Cancer Center University of Louisville, Louisville, USA; 3 Department of Pharmacology and Toxicology, University of Louisville, Louisville, USA; 4 Owensboro Cancer Research Program, Owensboro, USA; 5 Institute of Infectious Disease and Molecular Medicine, University of Cape Town, Cape Town, South Africa

## Abstract

**Background:**

A variety of interactions between up to three different movement proteins (MPs), the coat protein (CP) and genomic DNA mediate the inter- and intra-cellular movement of geminiviruses in the genus *Begomovirus*. Although movement of viruses in the genus *Mastrevirus *is less well characterized, direct interactions between a single MP and the CP of these viruses is also clearly involved in both intra- and intercellular trafficking of virus genomic DNA. However, it is currently unknown how specific these MP-CP interactions are, nor how disruption of these interactions might impact on virus viability.

**Results:**

Using chimaeric genomes of two strains of Maize streak virus (MSV) we adopted a genetic approach to investigate the gross biological effects of interfering with interactions between virus MP and CP homologues derived from genetically distinct MSV isolates. MP and CP genes were reciprocally exchanged, individually and in pairs, between maize (MSV-Kom)- and *Setaria *sp. (MSV-Set)-adapted isolates sharing 78% genome-wide sequence identity. All chimaeras were infectious in *Zea mays *c.v. Jubilee and were characterized in terms of symptomatology and infection efficiency. Compared with their parental viruses, all the chimaeras were attenuated in symptom severity, infection efficiency, and the rate at which symptoms appeared. The exchange of individual MP and CP genes resulted in lower infection efficiency and reduced symptom severity in comparison with exchanges of matched MP-CP pairs.

**Conclusion:**

Specific interactions between the mastrevirus MP and CP genes themselves and/or their expression products are important determinants of infection efficiency, rate of symptom development and symptom severity.

## Background

Mutation studies are often employed in attempts to identify the genetic basis of important aspects of a pathogen's phenotype. For example, in order to understand the genomic determinants of pathogenicity, genetic elements may be altered in, deleted from, or exchanged between virulent and benign pathogen isolates. During the last two decades, molecular biologists studying the ssDNA geminiviruses (family: *Geminiviridae*) have made extensive use of intra- and intergeneric genetic exchange in a wide variety of experiments. Briddon *et al*. [1] replaced the coat protein gene of the whitefly-transmitted African cassava mosaic begomovirus (ACMV) with that of beet curly top curtovirus (BCTV) and successfully transmitted the recombinant ACMV via the BCTV-specific leafhopper vector *Circulifer renellus *(Baker), thereby demonstrating that insect vector specificity for geminiviruses is determined by the coat protein. Similarly, Liu *et al*. [2] constructed chimaeras of the dicot-infecting mastrevirus bean yellow dwarf virus and the very distantly related monocot infecting mastrevirus maize streak virus (MSV) with the aim of identifying host specificity determinants. Although none of these chimaeras were able to systemically infect host plants of either parental virus, the study demonstrated the importance of intragenomic interactions in mastreviruses, and exposed the consequent limitations of genetic swaps between such diverse members of the genus. Subsequently, Martin and Rybicki [3] used chimaeras of closely related MSV variants to demonstrate that the primary sequence determinants of pathogenicity in maize resided in the MSV movement (MP) and coat protein (CP) genes.

Among the bipartite begomoviruses pseudorecombination of A and B components has formed the basis of many useful studies illuminating various *trans*-acting functions important in replication [4,5], symptom development [6], and *in planta *virus movement [7].

Findings from a large number of studies have led to a fairly detailed model of bipartite begomovirus movement [8] involving interactions between viral DNA and the nuclear shuttle protein (NSP, encoded by ORF BV1), and between a viral DNA-NSP complex and the movement protein (MP, encoded by ORF BC1). There is good *in vitro *evidence for analogous interactions involving the MP (encoded by ORF V2 or *mp*) and coat protein (CP; encoded by ORF V1 or *cp*) of mastreviruses [9-12] but the specificity of these interactions and their impact on MSV pathogenicity have not yet been fully explored in the context of natural infections. We felt that it should be possible to employ genetic complementation to illustrate the functional relevance of sequence-specific interactions between the mastrevirus MP and CP.

In the small and informationally compact MSV genome, deletion or inactivation of any genes results in asymptomatic infections or loss of infectivity [13,14] and in some cases even small alterations in coding or intergenic regions have resulted in dramatic attenuation of virulence [13,15-23]. While some of these mutations obviously altered amino acid sequences [13,22] or disrupted conserved DNA sequences required for replication or transcription [19,20,23] the deleterious effects of other mutations has been more difficult to explain [16,21]. In one instance, 11 of the 14 N-terminal amino acids of the MSV MP were altered without causing a noticeable loss of virulence [13] but this is an exceptional case in the literature. Notwithstanding the apparent fragility of mastreviruses in the face of mutation, we reasoned that relatively substantial genetic changes might be tolerated if effected via the exchange of homologous genomic modules, rather than through the introduction of isolated point mutations or deletions. We took an ambitious stance and set out to exchange the virion-sense ORFs between two of the most divergent MSV strains known – MSV-Kom and MSV-Set.

MSV-Kom and MSV-Set are both well characterized in terms of their host ranges, transmission dynamics, and symptomatology: both infect susceptible maize varieties and are transmitted by the same leafhopper vector, *Cicadulina mbila *Naudé [24]. The viruses share 78% nucleotide sequence identity overall, with their *mp *and *cp *genes respectively sharing 80% and 79% nucleotide sequence identity. MSV-Kom is an isolate of the MSV-A strain, which is the predominant MSV strain infecting maize in Africa [25,26]. MSV-Set, on the other hand, is one of only two characterized representatives of the Setaria-adapted MSV-C strain, and produces considerably milder symptoms in maize than does MSV-Kom [24]. Here we describe the construction of a series of six infectious MSV-Kom/MSV-Set chimaeric genomes comprising all the possible combinations of parental virus, *mp *and *cp *regions. Both parental viruses and all six recombinant viruses were assessed in terms of infectivity and symptomatology, and evidence of biologically-important specific interactions between the MSV MP and CP is presented.

## Results

### Viability of chimaeric genomes

To facilitate the exchange of the *mp *and *cp *genome regions, PCR-mediated mutagenesis was used to create NcoI restriction sites at the start codons of *cp *in the MSV-Kom and MSV-Set genomes (KNco and SNco respectively; [Additional file 1] [Additional file 2] [Additional file 3]); the cloned PCR products were sequenced to ensure that no unintentional mutations had been introduced (data not shown). The corresponding T→G mutations resulted in the substitution of alanine for serine at the second positions of the CP amino acid sequences [Additional file 2]. While these substitutions were expected to be conservative, mutant and wild-type viruses were compared by infecting Z. *mays *cv. Jubilee (a sweetcorn) to confirm that the *Nco*I mutation did not affect either infectivity or symptomatology in this host. Both NcoI mutants were indistinguishable from their wild-type counterparts in terms of their infectivity and the symptoms they produced (data not shown). In addition to KNco and SNco, all six chimaeric viruses produced symptoms in sweetcorn plants following agroinoculation.

Altogether, *mp *exchanges produced changes at 62 out of 320 nucleotide positions, resulting in the alteration of 22 out of 101 MP amino acid residues [Additional file 3]; switching the 697 bp *cp *led to 153 nucleotide changes affecting 39 out of 232 possible CP residues [Additional file 2]. Using a PAM250 substitution matrix [27] score of less than one as a guide, ten of the MP differences could be considered to be non-conservative, of which eight appear within the C-terminal quarter of the sequence. Using the same criterion, seventeen of the thirty-nine differences in the CP sequences represent non-conservative substitutions, none of which occurs among the fifty-eight C-terminal residues. Of the known splicing features in the *mp *[28], only the putative branch point sequence is different between MSV-Kom and -Set, but both variants comply with the requisite intron branch point consensus sequence (YUNAN) [29].

### Symptom severity and streak morphology

**Figure 1:**
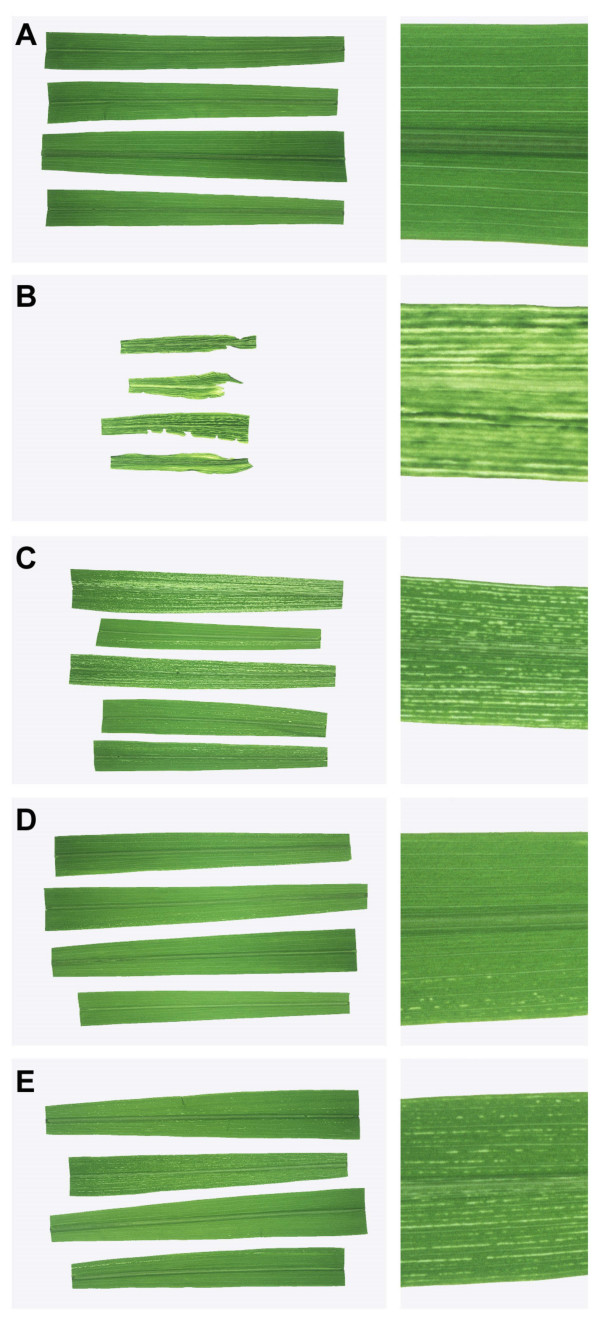
Streak symptoms produced by MSV-Kom-based constructs.

**Figure 2:**
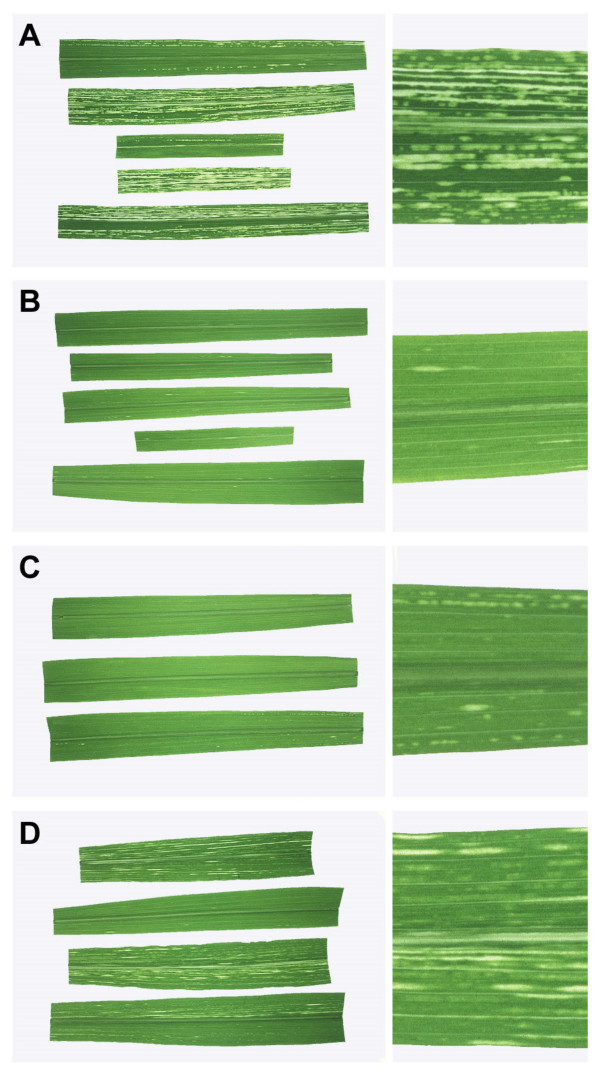
Streak symptoms produced by MSV-Set-based constructs.

Both symptom severity and streak character varied markedly among the viruses tested. In sweetcorn, KNco typically and consistently produced extensive, yellowish chlorotic streaks which, in extreme cases, were almost as wide as the leaf, and usually extended unbroken for several centimetres (Figure 1). In severe KNco infections, plants and leaves were noticeably stunted and in some cases leaves were malformed and curled. In contrast, the symptoms of SNco infection were milder in most respects (Figure 2): the total chlorotic area per leaf was smaller and more variable among SNco infected plants than among plants infected with KNco; SNco did not cause severe stunting, curling or malformation of infected leaves; SNco streaks tended to be shorter and narrower than those of KNco, resulting in a more stippled appearance. However, in one respect SNco appeared to be more pathogenic than KNco in that SNco caused more acute chlorosis, giving rise to whiter streaks. In severe instances the chlorotic tissue eventually disintegrated, leading to fine perforations in the leaves of some SNco-infected plants.

All the chimaeric daughter viruses displayed less virulence than either KNco or SNco (Table 1). With the notable exception of the relatively severe symptoms of K-MP-S (Figure 1; see Table 1 for the meaning of chimaeric virus names), chimaeras containing unmatched *mp-cp *pairs (K-CP-S, Figure 1; S-MP-K and S-CP-K, Figure 2) produced the mildest symptoms: chlorotic lesions were confined to short, narrow streaks that were sparsely distributed across the leaf. In contrast, the two reciprocal chimaeras containing matched *mp-cp *pairs (K-MP-CP-S and S-MP-CP-K) were significantly more pathogenic, with S-MP-CP-K showing particularly severe streak symptoms.

**Table 1:** Naming and symptomatology of MSV-Kom and -Set chimaeras.

Virus	Origin of ORF: (MSV-**K**om/MSV-**S**et)		Streak colour: (**Y**ellow/**W**hite)	Symptom severity (1 = mild;10 = severe)
	*mp*	*cp*		
KNco	K	K	Y	10
K-MP-S	S	K	W	7
K-CP-S	K	S	Y	1
K-MP-CP-S	S	S	W	5
SNco	S	S	W	9
S-MP-K	K	S	Y	2
S-CP-K	S	K	W	3
S-MP-CP-K	K	K	Y	8

As with the parental KNco and SNco viruses, the lesions produced by the chimaeras differed in their degree of chlorosis, and could be classified as either yellow (MSV-Kom-like) or white (MSV-Set-like; Table 1). Chimaeric viruses carrying the MSV-Kom *mp *– K-CP-S, S-MP-K, and S-MP-CP-K – produced yellowish streaks, while those carrying the MSV-Set *mp *– K-MP-S, K-MP-CP-S, and S-CP-K – produced streaks that were more severely chlorotic and were correspondingly distinctly white.

### Infection efficiencies and rates of symptom development

To provide additional indications of viral fitness, infectivity and the rate of symptom appearance were determined for each virus by inoculating plants with agroinfectious constructs and then monitoring them for symptom development.

**Figure 3:**
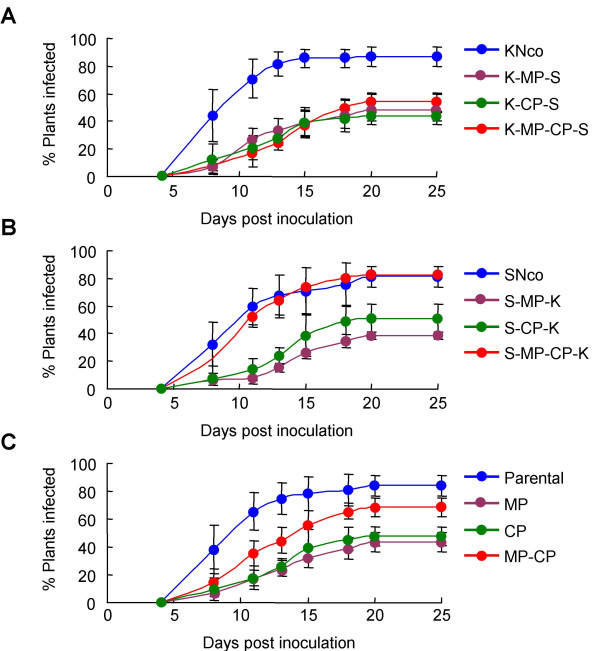
Average infection rates of chimaeras compared with parental viruses. A – Chimaeras based on MSV-Kom; B – chimaeras based on MSV-Set; C – averaged, combined data for MSV-Kom and -Set based chimaeras. Error bars represent standard deviations.

Figure 3 shows the rate at which symptoms appeared in plants following agroinoculation with each virus. As expected, plants inoculated with SNco developed symptoms slightly later than did plants inoculated with KNco, but both viruses showed very few new infections after fifteen days post inoculation (dpi). Over the course of twenty-five days, SNco infected a somewhat smaller percentage of plants than did KNco [SNco, 81% ± 7% (mean ± SD) ; KNco, 87% ± 7%].

Despite considerable differences in symptom severity, inoculation with all of the KNco-based chimaeras gave rise to symptomatic plants at similar rates, which were much slower than that of either parental virus. Accordingly, these chimaeras infected a significantly smaller percentage of plants over a twenty day period than did either parent: K-MP-S, 48% ± 11%; K-CP-S, 44% ± 2%; and K-MP-CP-S, 54% ± 7%. No new infections were observed later than 25 dpi (data not shown).

Compared with the KNco-based chimaeras, infection rates among SNco-based viruses were more distinct: while S-MP-K and S-CP-K both showed reductions in infectivity similar to those seen in KNco-based chimaeras, plants inoculated with S-MP-CP-K became symptomatic at a similar rate to those inoculated with SNco. Of all the viruses in this study, S-MP-K infected the lowest percentage of plants (38% ± 2%), while S-CP-K appeared to be slightly more infectious (51% ± 12% of plants infected).

**Figure 4:**
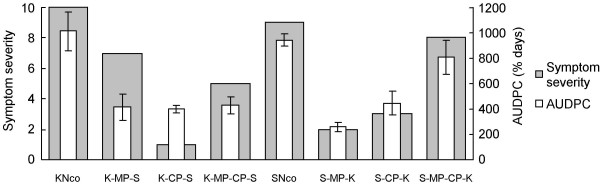
Average area under the disease progress curve and symptom severity for each chimaera, compared with the parental viruses KNco and SNco. Error bars represent standard deviations. Symptom severity and AUDPC are positively correlated (R^2 ^= 0.75; P = 0.005).

The fitness of each of the chimaeras and their parental viruses is summarized in Figure 4, which shows the average area under the disease progress curve (AUDPC) and symptom severity for each agroinfectious construct. The chimaera comprising both MSV-Kom *mp *and *cp *exchanged into the MSV-Set genome showed the highest AUDPC of all the chimaeric constructs, achieving 80% and 85% of the AUDPC figures of KNco and SNco respectively. The remaining recombinant viruses all showed large reductions in infectivity, resulting in AUDPC figures less than half that of either parent. Symptom severity followed a similar pattern, except that K-MP-S appeared to be relatively more virulent and K-CP-S relatively less virulent than the infectivity data would suggest.

## Discussion

### MSV-Kom/MSV-Set chimaeric viruses are infectious

Few directed mutagenesis studies of mastreviruses have been reported, and of these, most have focused on knocking out entire genes with the aim of establishing their functions [13,14,30]. Where genetic variants of MSV have been compared, relatively small differences – such as single nucleotide substitutions – have often been found to be responsible for rather large phenotypic disparities [16-18,22]. Considering the apparent sensitivity of MSV to mutation and the inability of similar BeYDV/MSV chimaeras to produce systemic infections [2] it may seem surprising that all the MSV-Kom/MSV-Set chimaeras described here are infectious. However, it should be borne in mind that directed mutagenesis studies are usually aimed at interrogating sequences suspected or known to be functionally critical, and neutral mutations are unlikely to be specifically reported because they are not generally considered interesting. Moreover, while numerous, the effective "point mutations" made in this study are of a special type – they comprise a set of mutations known to function well together within the context of the original, parental virus. That is to say, because entire homologous ORFs were exchanged, no intra-ORF or intra-protein interactions were disrupted in the chimaeras.

### Determinants of chlorotic severity

In view of the number of known – as well as the many likely but as yet unknown – *trans*-acting mechanisms engaged in functions such as gene regulation, virus replication, virus movement, virus-host interactions, *et cetera*, it is unsurprising that simple correlations between genotype and phenotype were not observed among the chimaeras described here: neither *mp *nor* cp*, either individually or together, could be considered wholly responsible for an MSV-Kom- or Set-like phenotype. However, the data do suggest that *mp *is a determinant of the severity of chlorosis, with the MSV-Set *mp *inducing whiter chlorotic streaks than that of MSV-Kom (Figure 1 and 2; Table 1). The movement protein gene not only encodes the MP, but also comprises an intron [Additional file 3] which is thought to affect *cp *expression levels [28] so it is also possible that variations in chlorosis were mediated via differences in *cp *expression.

Since the underlying causes of the chlorosis seen in MSV-infected tissue are not known, the significance of the varying degrees of chlorosis noted here is not obvious. It has been shown that chlorosis occurs only in infected cells [31] so it seems evident that the causal link between virus and chlorosis is fairly direct. One possibility is that chlorosis arises from the simple toxicity of one or more viral gene products. It is known that the MSV MP seems sufficiently toxic in *E. coli *to require strict control of expression for the generation of stable MP-expressing recombinants (personal observation, and personal communication from M.I. Boulton). Expression of geminivirus MP in transgenic plants can negatively affect plant development, necessitating the use of defective MP transgenes to regenerate healthy plants [32,33]. In contrast, geminivirus CPs have readily been over-expressed both in transgenic plants (TYLCV) [34] and in *E. coli *(MSV) [11] with no apparent adverse effects; the CP is unlikely to be inherently toxic. Thus, one hypothesis is that MP causes chlorosis as a result of its toxicity, and that the MSV-Set MP is inherently more toxic than that of MSV-Kom in sweetcorn. Alternatively, it is possible that the MSV-Set and -Kom MPs are similarly toxic, but that the MSV-Set MP is expressed in higher concentrations than MSV-Kom MP.

A second hypothesis is that the MSV MP and/or CP modulate a hypersensitive response [35,36] (HR reviewed in [37]) or other innate defense pathway in infected cells, which results in chlorosis. Geminivirus CPs have distant but detectable homology to begomovirus nuclear shuttle proteins (NSPs)[38], and it is worth noting that NSPs have been shown to elicit the HR [39] and to interact specifically with membrane-localised receptor-like kinases that are likely to play a role in defense responses [40]. A number of possibilities then follow: (1) that the MSV-Set MP is a more potent elicitor – or attains higher concentrations – than that of MSV-Kom; (2) that the MSV-Kom MP is a more effective inhibitor of the defense response; or (3) that *mp *influences CP levels, which in turn modulates the hypothetical defense response.

### Movement and coat protein genes interact specifically to facilitate infection and symptom development

Symptom severity and infection efficiency were roughly correlated (Pearson's R^2 ^= 0.75, P = 0.005; Figure 4) although K-MP-S displayed relatively severe symptoms in relation to its infection efficiency, whereas K-CP-S displayed comparatively mild symptoms. In both parental backgrounds, the exchange of cognate *mp-cp *pairs rescued much of the fitness lost through single gene exchanges: K-MP-CP-S was considerably more infectious and pathogenic than K-CP-S; similarly, S-MP-CP-K was almost as infectious and virulent as SNco, whereas both S-MP-K and S-CP-K were drastically compromised in both respects.

These observations provide strong evidence in support of the importance of specific *mp-cp *interactions in natural infections of maize. Specific binding of MP and CP has been demonstrated *in vitro *and *in vivo *[18] and some progress has been made in drawing parallels between the mechanisms underlying MSV cell-to-cell movement and the rather more developed models of movement in begomoviruses. MSV CP is localized to the nucleus and facilitates nuclear transport of viral DNA [12] which may be analogous to the nuclear localization and/or shuttling functions performed by begomovirus CPs and/or NSPs [41,42]; and MSV MP is localized at the cell periphery and binds to CP [43], which is reminiscent of at least some begomovirus MPs that have been shown to associate with plasma membranes and cell walls [44,45] and to co-operate with NSP in moving viral DNA out of the nucleus to adjacent cells [46-48]. As others have noted, the roles that CP, NSP, and MP play in intra- and inter-cellular movement seem to differ somewhat among various geminiviruses [42,8] and a detailed model of mastrevirus movement has yet to be elucidated.

### Modularity of genetic elements

Although the idea of the modularity of genetic elements is inherent in the traditional concept of the gene, this notion of neatly delineated, modular genes has become increasingly blurred by the discovery of the myriad complex interactions governing gene expression and protein function. Thus it has become clear that relatively few genes act independently, while some phenotypic characters arise from intricate webs of highly specific interactions between numerous distinct genetic sequence elements. One might imagine that the structures of these genetic interaction networks define the boundaries of functional genetic modules, which may range in size from a few nucleotides in the case of some regulatory sequences to many megabases in the case of an entire genome. Here we present evidence that the *mp-cp *cassette may represent such a functional genetic module in mastreviruses.

## Conclusion

This study provides some interesting perspectives on the varying degrees of modularity among the genetic regions studied here – namely *mp, cp*, *mp-cp*, and the remainder of the MSV genome. The results imply that *mp *is modular with respect to the degree of chlorosis it elicits in infected tissues, but not with respect to infection efficiency or chlorotic area. Similarly, exchanging *cp *alone was insufficient to maintain high infection rates or extensive virus movement. In contrast, the *mp-cp *cassette behaved in a far more modular fashion, in that exchanging this region had a relatively small effect on both virus infectivity and, judging from symptom development, *in planta *virus movement; it follows that the remainder of the MSV genome reflected the same degree of modularity.

## Methods

### Virus isolates, plasmids, bacterial strains, enzymes, and maize genotypes

The cloning vectors pBluescriptSK+ (pSK+; Stratagene, La Jolla, CA) and pUC19 (Stratagene), and the RecA- Escherichia coli strains DH5α and JM109 were used in all standard cloning procedures. The E. coli/A. tumefaciens binary vector pBI121 (Clontech, CA, U.S.A.) was used to produce agroinfectious DNA constructs, and the Agrobacterium tumefaciens strain C58C1 [pMP90][49] was used for all agroinoculations. Restriction enzymes and DNA ligase were obtained from a variety of commercial suppliers and were used according to the manufacturers' instructions. Sweetcorn maize cv. Jubilee seeds were purchased from Starke Ayres nursery (Rosebank, Cape Town, South Africa). The construction of MSV-Kom and MSV-Set full genome clones (pKom and pSet) and agroinfectious clones (in pBI121) has been described elsewhere [24].

### Construction of infectious chimaeric viral genomes

The construction of clones and agroinfectious constructs for the chimaeras K-MP-S, K-CP-S, S-MP-K and S-CP-K has been briefly described elsewhere [50] but will be fully explained here. Six chimaeric viral genomes were constructed by reciprocally exchanging *mp *and *cp *either singly, or in pairs, between pMSV-Kom and pMSV-Set. To facilitate these exchanges, it was necessary to first introduce *Nco*I restriction sites near the *cp *start codons of pKom and pSet to produce pKNco and pSNco respectively. This was achieved by inducing T→G transversions at nt. positions 468 and 471 (relative to the virion strand *ori*) in the MSV-Kom and -Set genomes respectively.

pKNco and pSNco were completely digested with *Pst*I and partially digested with *Nco*I. Fragments of both plasmids approximately 0.33 kbp, 0.69 kbp, and 4.4 kbp in size (respectively referred to as K1, K2, and K3 for pKNco and S1, S2, and S3 for pSNco) were purified by agarose gel electrophoresis. Ligations were performed using equimolar quantities of the purified fragments from pKNco and pSNco, using the following combinations of fragments: 1) S1, K2, K3; 2) K1, S2, K3; 3) S1, S2, K3; 4) K1, S2, S3; 5) S1, K2, S3; 6) K1, K2, S3. These six ligations respectively yielded the clones (1) pK-MP-S; (2) pK-CP-S; (3) pK-MP-CP-S; (4) pS-MP-K; (5) pS-CP-K; and (6) pS-MP-CP-K.

Agroinfectious clones of KNco, K-MP-S, K-CP-S, K-MP-CP-S, SNco, S-MP-K, S-CP-K, and S-MP-CP-K [Additional file 1] were constructed as described previously for pSet and pKom [24].

### Agroinoculation and Analysis of symptoms

Agroinfectious clones were used to transform *A. tumefaciens *C58C1 [pMP90], and agrinoculated into three day old maize seedlings as has been described previously [51].

Each inoculated plant was inspected for symptoms of virus infection regularly until twenty days post inoculation (dpi; day 0 = day of inoculation) and thereafter every week until 45 dpi. Symptoms on the first emergent leaf were disregarded to avoid confusion with physical damage inflicted during injection. Plants that did not survive agroinoculation and subsequent planting were disregarded for all subsequent analyses. The percentage of symptomatic plants was used as a measure of infection efficiency and disease progression. Calculations of area under the disease progress curve (AUDPC) were performed using the simple trapezoidal rule for calculating areas. By assessing chlorotic areas, stunting, curling and malformation of photographed leaves we subjectively ranked and scored then on a scale of 1 to 10, with 1 being the mildest and 10 the most severe symptoms.

## List of abbreviations used

ACMV: African casava mosaic virus; AUDPC: Area under the disease progress curve; BCTV: Beet curly top virus; BeYDV: Bean yellow dwarf virus; CP: Coat protein; cp: Coat protein gene; dpi: Days post infection; HR: Hypersensitive response; MP: movement protein; mp: movement protein gene; MSV: Maize streak virus; NSP: Nuclear shuttle protein; ORF: Open reading frame; PCR: Polymerase chain reaction; SD: Standard deviation; TYLCV: Tomato yellow leaf curl virus.

## Competing interests

The authors declare that they have no competing interests.

## Authors' contributions

EvdW conceived the study, carried out the experiments, and prepared the manuscript. KEP conceived the study, helped construct chimaeric genomes and supervised the study. DPM helped prepare the manuscript. EPR supervised the study, secured funding for its execution and helped prepare the manuscript. All authors read and approved the final manuscript.

## Supplementary Material

Additional file 1 MSV-Kom/MSV-Set chimaeric infectious plasmid constructs. Vector sequences are not shown. Arrows indicate ORFs in the direction of transcription; MSV-Kom sequences are shown in black and MSV-Set sequences in grey. Complete genomes are bounded by vertical dashed lines. The repetition of the stem-loop structure in the LIR allows replicational release of the genomes upon agroinfection. Restriction sites are indicated by▲; B = *Bam*HI, E = *Eco*RI, N = *Nco*I, X = *Xba*I. * The NcoI sites between *mp *and *cp *were introduced via PCR-mediated mutagenesis. Click here for file

Additional file 2 Nucleotide and amino acid changes resulting from coat protein (CP) gene exchanges. Upper line: MSV-Kom CP region, nucleotide sequence with corresponding CP amino acid sequence below (unique residues in bold, red typeface). Lower line: MSV-Set CP region, nucleotide sequence with corresponding CP amino acid sequence below (unique residues are in bold, green typeface). Nucleotide differences are indicated with ○ and amino acid differences with ◇ or ♦; amino acid differences with scores < 1 in the PAM250 substitution matrix are marked with ♦; * indicates a stop codon. The predicted nuclear localization signal (Liu *et al*., 1999b) and DNA binding domain (Liu *et al*., 1997) are highlighted and labeled in the diagram. Restriction sites used for exchanging sequences are underlined. The S→A mutation resulting from the introduction of the NcoI site is shown with †. Total nucleotide changes in the exchanged region: 153/697 positions (22.0%). Total amino acid changes in the exchanged region: 39/232 positions (16.8%). Click here for file

Additional file 3 Nucleotide and amino acid changes resulting from movement protein (MP) gene exchanges. Upper line: MSV-Kom *mp*, nucleotide sequence with corresponding MP amino acid sequence below (unique residues in bold, red typeface). Lower line: MSV-Set *mp*, nucleotide sequence with corresponding MP amino acid sequence below (unique residues are in bold, green typeface). Nucleotide differences are indicated with ○ and amino acid differences with ◇ or ♦; amino acid differences with scores < 1 in the PAM250 substitution matrix are marked with ♦; * indicates a stop codon. The predicted trans-membrane domain (Boulton *et al*., 1993) and splicing features (Wright *et al*., 1997) are highlighted and labeled in the diagram. Restriction sites used for exchanging sequences are underlined. Total nucleotide changes in exchanged region: 62/320 positions (19.4%). Total nucleotide changes in ORF: 60/306 positions (19.6%). Total amino acid changes: 22/101 positions (21.8%). Click here for file

## References

[B1] Briddon RW, Pinner MS, Stanley J, Markham PG (1990). Geminivirus coat protein gene replacement alters insect specificity. Virology.

[B2] Liu L, Pinner MS, Davies JW, Stanley J (1999). Adaptation of the geminivirus bean yellow dwarf virus to dicotyledonous hosts involves both virion-sense and complementary-sense genes. J Gen Virol.

[B3] Martin DP, Rybicki EP (2002). Investigation of Maize streak virus pathogenicity determinants using chimaeric genomes. Virology.

[B4] Lazarowitz SG, Wu LC, Rogers SG, Elmer JS (1992). Sequence-specific interaction with the viral AL1 protein identifies a geminivirus DNA replication origin. Plant Cell.

[B5] Frischmuth T, Roberts S, von Arnim A, Stanley J (1993). Specificity of bipartite geminivirus movement proteins. Virology.

[B6] von Arnim A, Stanley J (1992). Determinants of tomato golden mosaic virus symptom development located on DNA B. Virology.

[B7] von Arnim A, Stanley J (1992). Inhibition of African cassava mosaic virus systemic infection by a movement protein from the related geminivirus tomato golden mosaic virus. Virology.

[B8] Hehnle S, Wege C, Jeske H (2004). Interaction of DNA with the movement proteins of geminiviruses revisited. J Virol.

[B9] Boulton MI, Pallaghy CK, Chatani M, MacFarlane S, Davies JW (1993). Replication of maize streak virus mutants in maize protoplasts: evidence for a movement protein. Virology.

[B10] Dickinson VJ, Halder J, Woolston CJ (1996). The product of maize streak virus ORF V1 is associated with secondary plasmodesmata and is first detected with the onset of viral lesions. Virology.

[B11] Liu H, Boulton MI, Davies JW (1997). Maize streak virus coat protein binds single- and double-stranded DNA in vitro. J Gen Virol.

[B12] Liu H, Boulton MI, Thomas CL, Prior DA, Oparka KJ, Davies JW (1999). Maize streak virus coat protein is karyophyllic and facilitates nuclear transport of viral DNA. Mol Plant Microbe Interact.

[B13] Boulton MI, Steinkellner H, Donson J, Markham PG, King DI, Davies JW (1989). Mutational analysis of the virion-sense genes of maize streak virus. J Gen Virol.

[B14] Lazarowitz SG, Pinder AJ, Damsteegt VD, Rogers SG (1989). Maize streak virus genes essential for systemic spread and symptom development. EMBO J.

[B15] Shen WH, Hohn B (1991). Mutational analysis of the small intergenic region of maize streak virus. Virology.

[B16] Boulton MI, King DI, Donson J, Davies JW (1991). Point substitution in a promoter-like region and the V1 gene affect the host range and symptoms of maize streak virus. Virology.

[B17] Boulton MI, King DI, Markham PG, Pinner MS, Davies JW (1991). Host range and symptoms are determined by specific domains of the maize streak virus genome. Virology.

[B18] Liu H, Lucy A, Davies JW, Boulton MI (2001). A single amino acid change in the coat protein of Maize streak virus abolishes systemic infection, but not interaction with viral DNA or movement protein. Mol Plant Path.

[B19] Schneider M, Jarchow E, Hohn B (1992). Mutational analysis of the 'conserved region' of maize streak virus suggests its involvement in replication. Plant Mol Biol.

[B20] Schnippenkoetter WH, Martin DP, Willment JA, Rybicki EP (2001). Forced recombination between distinct strains of Maize streak virus. J Gen Virol.

[B21] Shepherd DN, Martin DP, Varsani A, Thomson JA, Rybicki EP, Klump HH (2006). Restoration of native folding of single-stranded DNA sequences through reverse mutations: an indication of a new epigenetic mechanism. Arch Biochem Biophys.

[B22] Shepherd DN, Martin DP, McGivern DR, Boulton MI, Thomson JA, Rybicki EP (2005). A three-nucleotide mutation altering the Maize streak virus Rep pRBR-interaction motif reduces symptom severity in maize and partially reverts at high frequency without restoring pRBR-Rep binding. J Gen Virol.

[B23] Willment JA, Martin DP, Palmer KE, Schnippenkoetter WH, Shepherd DN, Rybicki EP (2007). Identification of long intergenic region sequences involved in maize streak virus replication. J Gen Virol.

[B24] Schnippenkoetter WH, Martin DP, Hughes FL, Fyvie M, Willment JA, James D, von Wechmar MB, Rybicki EP (2001). The relative infectivities and genomic characterisation of three distinct mastreviruses from South Africa. Arch Virol.

[B25] Briddon RW, Lunness P, Chamberlin LC, Markham PG (1994). Analysis of the genetic variability of maize streak virus. Virus Genes.

[B26] Martin DP, Willment JA, Billharz R, Velders R, Odhiambo B, Njuguna J, James D, Rybicki EP (2001). Sequence diversity and virulence in Zea mays of Maize streak virus isolates. Virology.

[B27] Schwartz RM, Dayhoff MO (1979). Protein and nucleic acid sequence data and phylogeny. Science.

[B28] Wright EA, Heckel T, Groenendijk J, Davies JW, Boulton MI (1997). Splicing features in maize streak virus virion- and complementary-sense gene expression. Plant J.

[B29] Simpson CG, Clark G, Davidson D, Smith P, Brown JW (1996). Mutation of putative branchpoint consensus sequences in plant introns reduces splicing efficiency. Plant J.

[B30] Liu L, Davies JW, Stanley J (1998). Mutational analysis of bean yellow dwarf virus, a geminivirus of the genus Mastrevirus that is adapted to dicotyledonous plants. J Gen Virol.

[B31] Lucy AP, Boulton MI, Davies JW (1996). Tissue specificity of Zea mays infection by maize streak virus. Mol Plant Microbe Interact.

[B32] Hou YM, Sanders R, Ursin VM, Gilberston RL (2000). Transgenic plants expressing geminivirus movement proteins: Abnormal phenotypes and delayed infection by Tomato mottle virus in transgenic tomatoes expressing the Bean dwarf mosaic virus BV1 or BC1 proteins. Mol Plant Microbe Interact.

[B33] Covey SN, Al-Kaff NS (2000). Plant DNA viruses and gene silencing. Plant Mol Biol.

[B34] Kunik T, Salomon R, Zamir D, Navot N, Zeidan M, Michelson I, Gafni Y, Czosnek H (1994). Transgenic tomato plants expressing the tomato yellow leaf curl virus capsid protein are resistant to the virus. Biotechnology.

[B35] Van Wezel R, Dong X, Blake P, Stanley J, Hong Y (2002). Differential roles of geminivirus Rep and AC 4(C 4) in the induction of necrosis in *Nicotiana benthamiana*. Molecular Plant Pathology.

[B36] Hong Y, Stanley J, van Wezel R (2003). Novel system for the simultaneous analysis of geminivirus DNA replication and plant interactions in Nicotiana benthamiana. J Virol.

[B37] Kiraly L, Barna B, Kiraly Z (2007). Plant Resistance to Pathogen Infection: Forms and Mechanisms of Innate and Acquired Resistance. Journal of Phytopathology.

[B38] Rybicki EP (1994). A phylogenetic and evolutionary justification for three genera of Geminiviridae. Arch Virol.

[B39] Garrido-Ramirez ER, Sudarshana MR, Lucas WJ, Gilbertson RL (2000). Bean dwarf mosaic virus BV1 protein is a determinant of the hypersensitive response and avirulence in Phaseolus vulgaris. Mol Plant Microbe Interact.

[B40] Fontes EP, Santos AA, Luz DF, Waclawovsky AJ, Chory J (2004). The geminivirus nuclear shuttle protein is a virulence factor that suppresses transmembrane receptor kinase activity. Genes Dev.

[B41] Lazarowitz SG, Beachy RN (1999). Viral movement proteins as probes for intracellular and intercellular trafficking in plants. Plant Cell.

[B42] Gafni Y, Epel BL (2002). The role of host and viral proteins in intra- and inter-cellular trafficking of geminiviruses. Phys Mol Plant Path.

[B43] Kotlizky G, Boulton MI, Pitaksutheepong C, Davies JW, Epel BL (2000). Intracellular and intercellular movement of maize streak geminivirus V1 and V2 proteins transiently expressed as green fluorescent protein fusions. Virology.

[B44] Pascal E, Sanderfoot AA, Ward BM, Medville R, Turgeon R, Lazarowitz SG (1994). The geminivirus BR1 movement protein binds single-stranded DNA and localizes to the cell nucleus. Plant Cell.

[B45] von Arnim A, Frischmuth T, Stanley J (1993). Detection and possible functions of African cassava mosaic virus DNA B gene products. Virology.

[B46] Sanderfoot AA, Lazarowitz SG (1996). Getting it together in plant virus movement: cooperative interactions between bipartite geminivirus movement proteins. Trends Cell Biol.

[B47] Sanderfoot AA, Lazarowitz SG (1995). Cooperation in viral movement: The geminivirus BL1 movement protein interacts with BR1 and redirects it from the nucleus to the cell periphery. Plant Cell.

[B48] Ward BM, Medville R, Lazarowitz SG, Turgeon R (1997). The geminivirus BL1 movement protein is associated with endoplasmic reticulum-derived tubules in developing phloem cells. J Virol.

[B49] Koncz C, Schell J (1986). The promoter of TL-DNA gene 5 controls the tissue-specific expression of chimaeric genes carried by a novel type of *Agrobacterium *binary vector. Molecular and General Genetics.

[B50] Martin DP, Walt E van der, Posada D, Rybicki EP (2005). The evolutionary value of recombination is constrained by genome modularity. PLoS Genet.

[B51] Martin DP, Willment JA, Rybicki EP (1999). Evaluation of maize streak virus pathogenicity in differentially resistant Zea mays genotypes. Phytopathology.

