# Supplementary figures and images for: Viable chimaeric viruses confirm the biological importance of sequence specific maize streak virus movement protein and coat protein interactions
Source: Virol J. 2008 May 20;5:61. doi: 10.1186/1743-422X-5-61 (PMC2430021; doi:10.1186/1743-422X-5-61)

## Slide 1
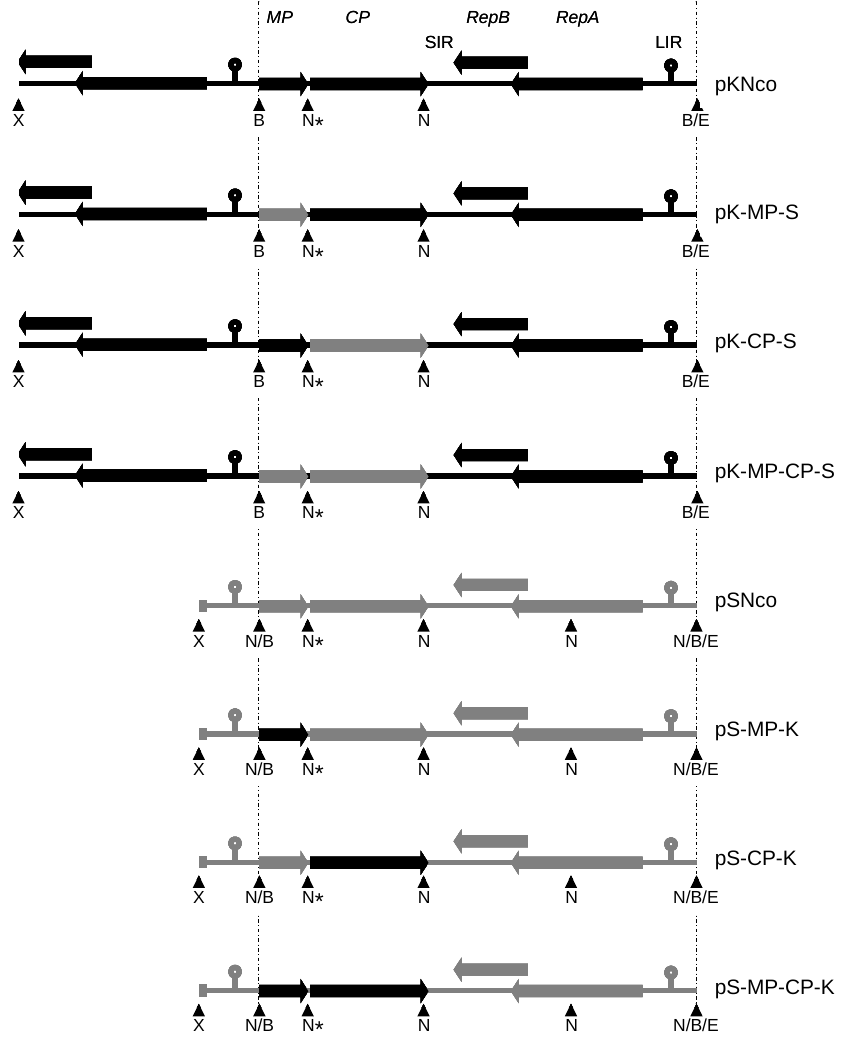

*
*
*
*
*
*
*
*
pKNco
pK-MP-S
pK-CP-S
pK-MP-CP-S
pSNco
pS-MP-K
pS-CP-K
pS-MP-CP-K

Supplement: Additional file 1 — MSV-Kom/MSV-Set chimaeric infectious plasmid constructs. Vector sequences are not shown. Arrows indicate ORFs in the direction of transcription; MSV-Kom sequences are shown in black and MSV-Set sequences in grey. Complete genomes are bounded by vertical dashed lines. The repetition of the stem-loop structure in the LIR allows replicational release of the genomes upon agroinfection. Restriction sites are indicated by▲; B = BamHI, E = EcoRI, N = NcoI, X = XbaI. * The NcoI sites between mp and cp were introduced via PCR-mediated mutagenesis. [file 1743-422X-5-61-S1.ppt]

## Slide 1
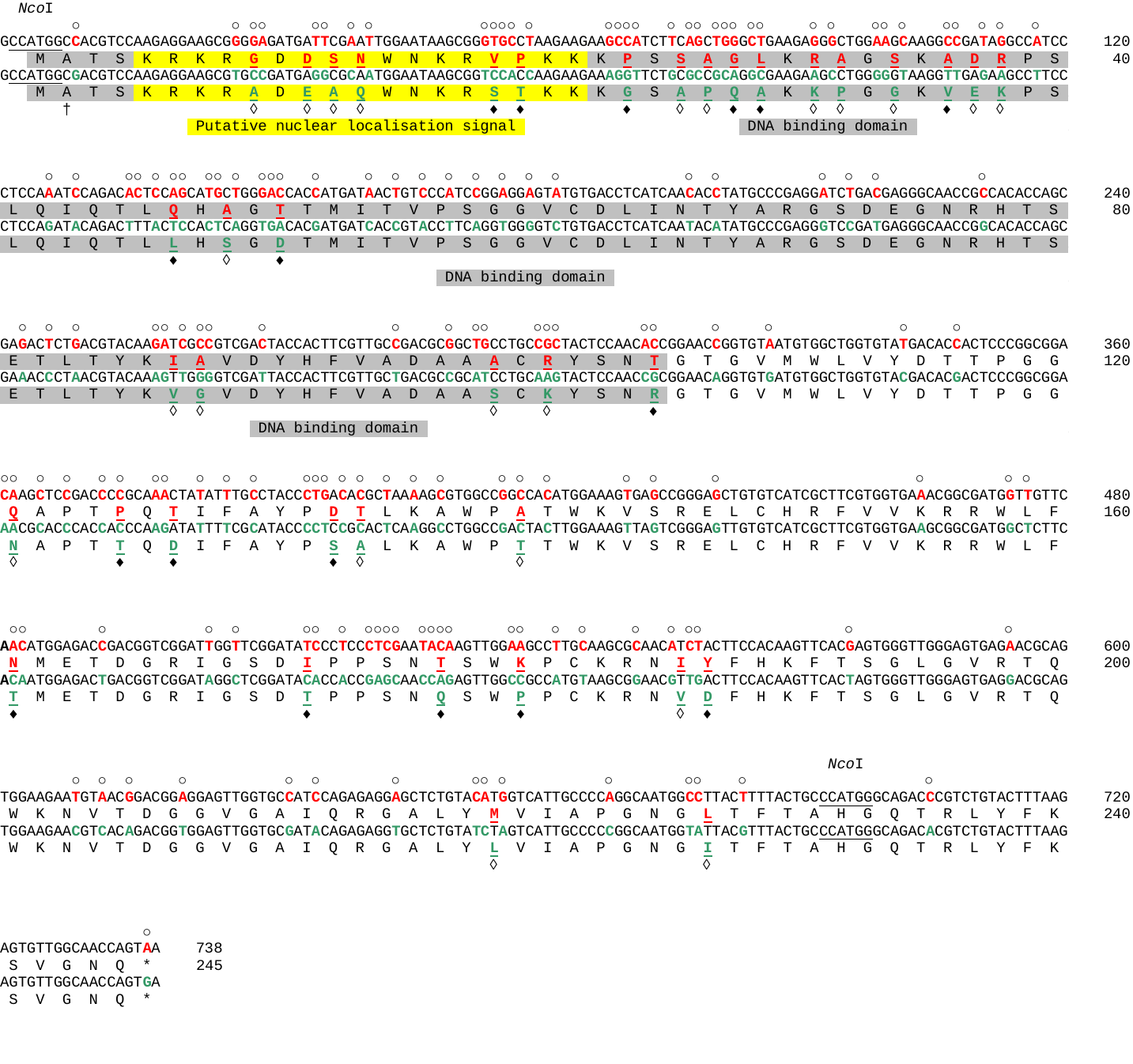

Supplement: Additional file 2 — Nucleotide and amino acid changes resulting from coat protein (CP) gene exchanges. Upper line: MSV-Kom CP region, nucleotide sequence with corresponding CP amino acid sequence below (unique residues in bold, red typeface). Lower line: MSV-Set CP region, nucleotide sequence with corresponding CP amino acid sequence below (unique residues are in bold, green typeface). Nucleotide differences are indicated with ○ and amino acid differences with ◇ or ♦; amino acid differences with scores < 1 in the PAM250 substitution matrix are marked with ♦; * indicates a stop codon. The predicted nuclear localization signal (Liu et al., 1999b) and DNA binding domain (Liu et al., 1997) are highlighted and labeled in the diagram. Restriction sites used for exchanging sequences are underlined. The S→A mutation resulting from the introduction of the NcoI site is shown with †. Total nucleotide changes in the exchanged region: 153/697 positions (22.0%). Total amino acid changes in the exchanged region: 39/232 positions (16.8%). [file 1743-422X-5-61-S2.ppt]

## Slide 1
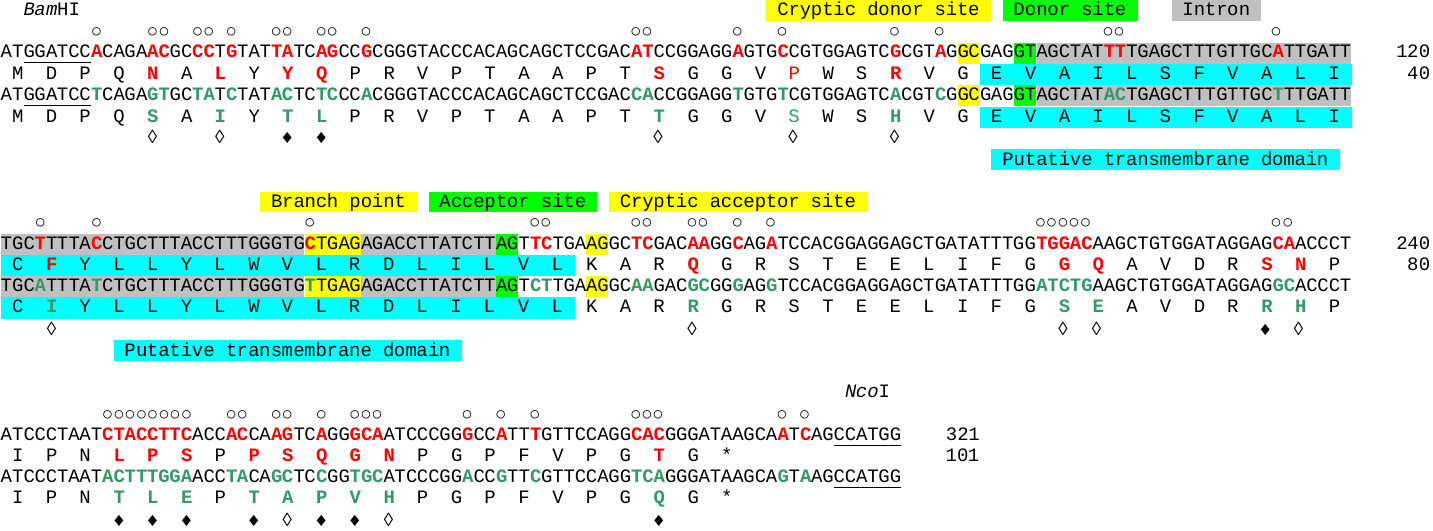

Supplement: Additional file 3 — Nucleotide and amino acid changes resulting from movement protein (MP) gene exchanges. Upper line: MSV-Kom mp, nucleotide sequence with corresponding MP amino acid sequence below (unique residues in bold, red typeface). Lower line: MSV-Set mp, nucleotide sequence with corresponding MP amino acid sequence below (unique residues are in bold, green typeface). Nucleotide differences are indicated with ○ and amino acid differences with ◇ or ♦; amino acid differences with scores < 1 in the PAM250 substitution matrix are marked with ♦; * indicates a stop codon. The predicted trans-membrane domain (Boulton et al., 1993) and splicing features (Wright et al., 1997) are highlighted and labeled in the diagram. Restriction sites used for exchanging sequences are underlined. Total nucleotide changes in exchanged region: 62/320 positions (19.4%). Total nucleotide changes in ORF: 60/306 positions (19.6%). Total amino acid changes: 22/101 positions (21.8%). [file 1743-422X-5-61-S3.ppt]
